# The effects of embodying wildlife in virtual reality on conservation behaviors

**DOI:** 10.1038/s41598-022-10268-y

**Published:** 2022-04-19

**Authors:** Daniel Pimentel, Sri Kalyanaraman

**Affiliations:** 1 Oregon Reality Lab, School of Journalism and Communication, University of Oregon, Portland, OR USA; 2 School of Journalism and Communications, University of Florida, Gainesville, FL USA

**Keywords:** Human behaviour, Psychology and behaviour

## Abstract

Efforts to mitigate environmental threats are often inversely related to the magnitude of casualty, human or otherwise. This “compassion fade” can be explained, in part, by differential processing of large- versus small-scale threats: it is difficult to form empathic connections with unfamiliar masses versus singular victims. Despite robust findings, little is known about how non-human casualty is processed, and what strategies override this bias. Across four experiments, we show how embodying threatened megafauna-Loggerhead sea turtles (*Caretta Caretta*)-using virtual reality can offset and reverse compassion fade. After observing compassion fade during exposure to non-human casualty in virtual reality (Study 1; *N* = 60), we then tested a custom virtual reality simulation designed to facilitate body transfer with a threatened Loggerhead sea turtle (Study 2; *N* = 98). Afterwards, a field experiment (Study 3; *N* = 90) testing the simulation with varied number of victims showed body transfer offset compassion fade. Lastly, a fourth study (*N* = 25) found that charitable giving among users embodying threatened wildlife was highest when exposed to one versus several victims, though this effect was reversed if victims were of a different species. The findings demonstrate how animal embodiment in virtual reality alters processing of environmental threats and non-human casualty, thereby influencing conservation outcomes.

## Introduction

Marine megafauna face a myriad of environmental threats (e.g., climate change), affecting the ocean’s biodiversity, condition, and functioning^1^. Despite the large scale of observed and projected marine biodiversity loss^2^, and scientific consensus on the need to mitigate such impacts^3^, awareness alone cannot spur pro-conservation behavioral change at scale^4^. As Manfredo argues, “human cognition will adapt to environmental threats” (p. 6), and nohere is this more evident than in the processing of mass casualty, where sensitivity to fatalities diminishes as the number of victims increase^5^. This phenomenon, referred to as “compassion fade^6^,” or “psychic numbing^5^,” occurs due to differential processing of the victim(s), and is observed with both human^7^ and non-human^6^ casualty.

According to evolutionary psychology, altruism towards a suffering party is driven by empathy, or the extent to which one can share the emotional state of the target, and identify with them^8^. However, when faced with multiple victims, our capacity to infer and share a group’s affective states diminishes. Put differently, mass victims are nebulous social targets whose suffering elicits negative affect and demotivation of approach-and giving-behavior^9,10^. This desensitization leads to underreactions to threats^11^, and can be observed when comparing a single victim with as little as two victims of the same species^6^. Given the enormity of projected biodiversity loss associated with various environmental threats^12,13^, how can mass loss of life be communicated so as to encourage connectedness with non-human victims and aid in environmental mobilization?

As Darwin suggests, we as humans are impelled to minimize the suffering of others so as to subdue our own painful feelings^14^. The magnitude of an empathic response is contingent on affective and cognitive processes, such as concern for the other and the ability to conjure mental imagery of the target’s lived experience, respectively^15^. Extant literature suggests that engaging in narrative perspective-taking (NPT), or mentally imagining oneself in the perspective of a story character^16^, may offset indifference to mass casualty by spurring empathy for victims, a noted antecedent to altruistic behavior^17,18^. While NPT is increasingly used by environmental organizations to connect audiences with the plight of non-human victims, public apathy for large-scale environmental threats and their victims persists^19–22^.

NPT is limited in its capacity to spur empathy with mass victims due to various factors. First, NPT is sensitive to individual differences^23^, and its effects are dulled when victims and their threats are temporally or spatially distant^19^. Second, NPT is itself a difficult and cognitively taxing exercise^24^, especially when targets are non-human animals^25–27^. As extant literature suggests, basic emotions seen in animals do not map onto human categories^28,29^, a belief also held by laypeople for many species^30,31^. If empathy is aided when a person feels they belong to the same category (e.g., species) as the victim^32^, the genetic differences between human and beast limit the ability to infer an animal’s mental and emotional state, impeding NPT, empathy, and relatedness^33^. This ultimately begs the question: Can audiences come to see themselves as part of a non-human mass of victims? If so, can this experience offset compassion fade?

In “Being a Beast,” Charles Foster chronicles his time spent living with and as wildlife in their natural habitats (e.g., Deer, Otters), describing the empathic connections forged as a result of this visceral perspective-taking^34^. Yet, one need not live as wildlife to embody a species’ lived experiences. With immersive media technology, namely virtual reality (VR), individuals can use head-mounted displays (HMDs) and motion tracking sensors to swap real-world sensory inputs with those of a simulated virtual environment^35^. In other words, by simulating the lived experience of another being through this audiovisual gestalt, VR users can experience a sense of “being there” (spatial presence) in the VE^36^, as well as a sense of “being with” (social presence) other virtual bodies (avatars) in the VE^37^. This virtual perspective-taking ultimately contributes to empathy in various contexts^38^, in part because events in VR imprint on the user’s autobiographical memory as lived experiences^39^.

Within these virtual spaces users can embody an avatar, or a digital self-representation which allows for them to interact with other users and the environment itself. Moreover, the ability to control a virtual body can contribute towards an illusory sense of actually owning the virtual body. This sense of body transfer (BT) is achieved via sensorimotor contingencies^40^, namely audiovisual and haptic correspondence between the user’s virtual and physical experience. In other words, when a user’s virtual body movements synchronize to their real-world body movements (visuomotor representation), this cultivates BT^41–43^. Similarly, when haptic feedback is received onto a user’s body corresponding to events affecting their virtual body (visuotactile stimulation), this too contributes to BT^44,45^.

Investigations of BT in VR have largely focused on the antecedents and effects of BT with humanoid bodies^42,46^. However, human embodiment is inherently different than animal embodiment for two major reasons: differences in social identity and incompatibilities between human and animal anatomy. First, animals are largely void of social identity cues discernable by humans, which makes it difficult to discern how animal embodiment influences responses to other non-human avatars. Second, humans differ significantly from wildlife in terms of structure (e.g., number of limbs) and core biomechanics, including species capable of bipedal walking (e.g., chimpanzees)^47^. Moreover, while evidence suggests that BT with non-human^48^ and non-bipedal bodies is possible^49,50^, the individual and tandem contributions of visuomotor representation and visuotactile stimulation in driving BT with a non-human body remain largely unknown, as are the perceptual and behavioral implications.

If “minds profoundly reflect the bodies in which they are contained”^51^ (p. 167), animal embodiment (BT with a non-human body) should influence cognitive and affective processing of (virtual) events, namely threats and associated victims. Indeed, as embodied cognition (EC) theories postulate, processing of (virtual) information is highly dependent on characteristics of an individual’s (virtual) body and its interactions with the (virtual) world^52,53^. Extant literature on BT demonstrates that embodied perspective-taking influences cognitive^46,54^ and affective^55^ processing. Moreover, observing potential^56^ or actual harm^57^ to one’s virtual body elicits physiological threat responses akin to in vivo exposure. This is particularly important considering that more self-relevant threats spur urgency to mitigate them^58^, especially in the context of environmental risks^59^.

In sum, this investigation presents three major propositions that explain why BT with a threatened species should mitigate compassion fade responses to mass non-human casualty. First, since BT is characterized as a self-other overlap^60^, this merger between user and victim should contribute to empathic connections by creating the sense that they belong to the same category^32^. Second, because this merger results in a user perceiving themselves as part of the victimized group, BT should facilitate reciprocal altruism, or motivations to aid targets of a similar group^61^. Lastly, because threats experienced during embodiment are processed as lived experiences^39^, exposure to mass casualty should lead to greater perceptions of encountered threats as more severe, imminent, and self-relevant, factors which contribute to pro-environmental outcomes^58^.

## Methodological overview

Four separate experiments were conducted to examine how embodying non-human victims influences compassion fade and broad conservation outcomes. A 2-condition (victims: one vs. seven) controlled lab experiment in Study 1 explored whether compassion fade occurs when humans are exposed to non-human casualties in VR. Study 2 then sought to determine the requirements for BT with a non-human being (sea turtle), employing a 2 (visuomotor representation: representation vs. no representation) × 2 (visuotactile stimulation: stimulation vs. no stimulation) between-subjects experiment. After establishing the feasibility and effectiveness of BT with a Loggerhead sea turtle body in VR, Study 3 employed a 3-condition (victims: none/one/seven) between-subjects field experiment to examine whether animal embodiment influenced compassion fade. Finally, Study 4 employed a 2 (victims: one/seven) × 2 (victim species: same/different) between-subjects experiment to determine whether the effects of embodiment on compassion fade observed in Study 3 were contingent on victim similarity.

For all four studies, experimental protocols were approved by the Committee for the Protection of Human Subjects and an Insitutional Review Board. Moreover, all methods were carried out in accordance with relevant guidelines and regulations.

### Statistical analyses

All statistical analyses were performed using IBM SPSS. Assessment of mean differences in donation amount for Study 1 employed a two-tailed *t*-test. Subsequent studies employed one-way and two-way analyses of variance (ANOVAs). Moderation and mediation analyses were also conducted within SPSS through the use of the PROCESS path-analysis macro. Significance was set at 0.05. Lastly, across all analyses, assumptions of homogeneity were met.

### Study 1: compassion fade in immersive environments

#### Methods: study 1

To test the assumption that compassion fade can be observed during immersive depictions of mass casualty, a two-condition (victims: one/seven) between-subjects experiment was conducted. Participants (*N* = 60; *M*_*age*_ = 20.97) were paid $5 to experience and evaluate an immersive public service announcement (PSA) wherein threats to loggerhead sea turtles were discussed in a hospital setting alongside one or seven members of the species (victims). Of the 60 participants, 23.3% identified as male (*N*_*male*_ = 14), 76.7% identified as female (*N*_*female*_ = 46).

### Stimulus materials

**Figure 1:**
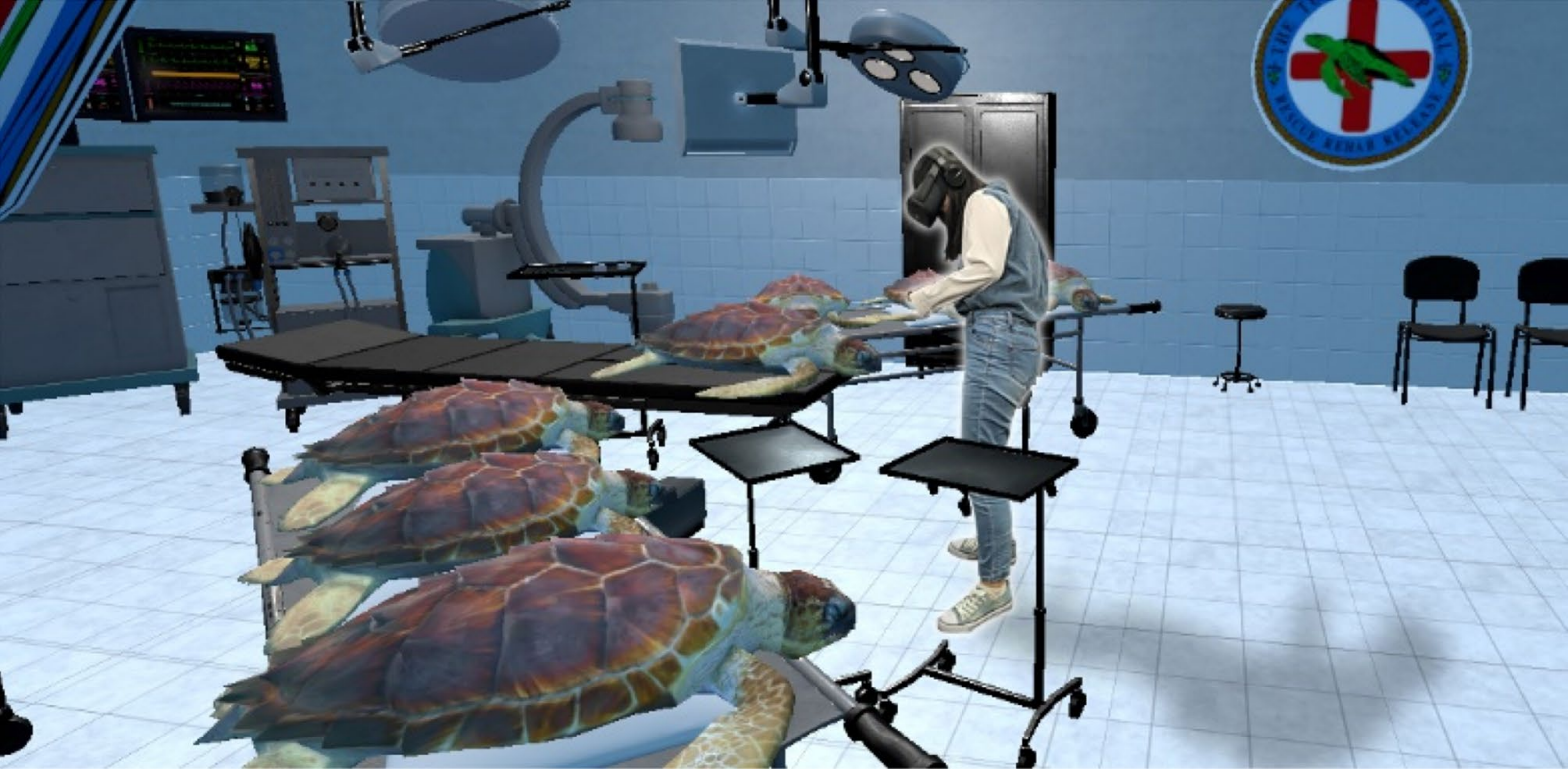
Visual representation of the Study 1 stimuli. A participant in the seven victims condition (left) inspects the virtual Loggerhead turtles.

To present mass versus singular wildlife casualty in a plausible context, a 2-min, room-scale VR PSA was created for use with the Oculus Quest HMD. Upon equipping the HMD, participants were able to explore a virtual sea turtle hospital modeled after an actual rehabilitation center in Florida. Upon entering the hospital, participants were either presented with one or seven injured Loggerhead sea turtles (see Fig. 1). Narration and animations then informed participants of various threats facing the species (e.g., ocean plastics, boat strikes), after which the PSA ended, leaving the participant free to remove their HMD.

### Procedures

Upon arriving at the lab, participants were informed that they would be asked to evaluate an environmental PSA using VR in exchange for $5. After providing informed consent, participants completed an online pre-questionnaire to assess demographic information (e.g., age, racial identity) as well as their level of environmentalism, which served as a control variable across all studies. Afterwards, participants were randomly assigned to one of the two PSAs. After receiving instructions on how to use the HMD, participants then completed the PSA, completed a final online survey assessing key dependent variables, and received payment.

### Dependent variables

To control for individual differences in environmental concern, environmentalism was measured using a 12-item 7-point Likert scale (1–7) adapted from previous work^62^. Measures of presence were assessed using spatial presence^63^ and social copresence^64^ scales adapted from previous work. Additionally, attitudes towards the PSA were assessed using a validated 12-item 7-point Likert scale (1–7) assessing the level of agreement with various adjectives describing the simulation^65^ (e.g., appealing, exciting). Pro-environmental behavioral intentions were also assessed via a 7-point Likert scale (1–7) asking participants to report the likelihood (very unlikely to very likely) that they would engage in five separate pro-environmental activities (e.g., Donate money) benefitting Sea Turtle conservation efforts. All scales exhibited moderate to high reliability (all Cronbach’s α > 0.75).

#### Measuring compassion fade

After completing the final online survey assessing key dependent variables, participants were escorted into an isolated room to receive payment. Once in the room, the researcher informed the participant that they would receive five (5) $1 bills in an envelope. Furthermore, participants were informed that they had the opportunity to donate all, some, or none of the $5 towards *The Turtle Hospital* by placing the desired donation amount in the envelope and leaving the envelope in a basket along with other sealed envelopes. This donation amount served as the key dependent variable.

To prevent demand effects, the researcher informed the participant that all donations are anonymous, and that the researcher would not know whether participants donated or not because the decision is made after the researcher steps out of the room. After exiting the room, participants were thanked and escorted out of the lab.

### Results: study 1

A series of Bonferroni corrected independent t-tests were conducted (see Table 1). Participants in the single victim condition donated, on average, more towards sea turtle conservation (*M* = 2.9, *SD* = 1.78) than those in the seven victims condition (*M* = 1.86, *SD* = 1.96), *t*(58) = 2.13, *P* = 0.03, *d* = 0.53. Thus, compassion fade effects were observed.

**Table 1:** Mean and standard deviation of dependent measures in Study 1.

Measure	Single Victim	Seven Victims	Difference
Attitudes	5.17 (1.24)	4.73 (.76)	*P* = *ns*
Spatial Presence	5.31 (1.25)	5.02 (1.27)	*P* = *ns*
Copresence	3.52 (1.01)	3.83 (.93)	*P* = *ns*
Donation	**$2.90** (**1.78)**	**$1.86** (**1.96)**	*P* **= .03**
Donation Intention	4.83 (1.64)	4.17 (1.28)	*P* = *ns*
Distance	**36.11** (**8.77)**	**30.26** (**4.62)**	*P* **= .005**

### Discussion: study 1

**Figure 2:**
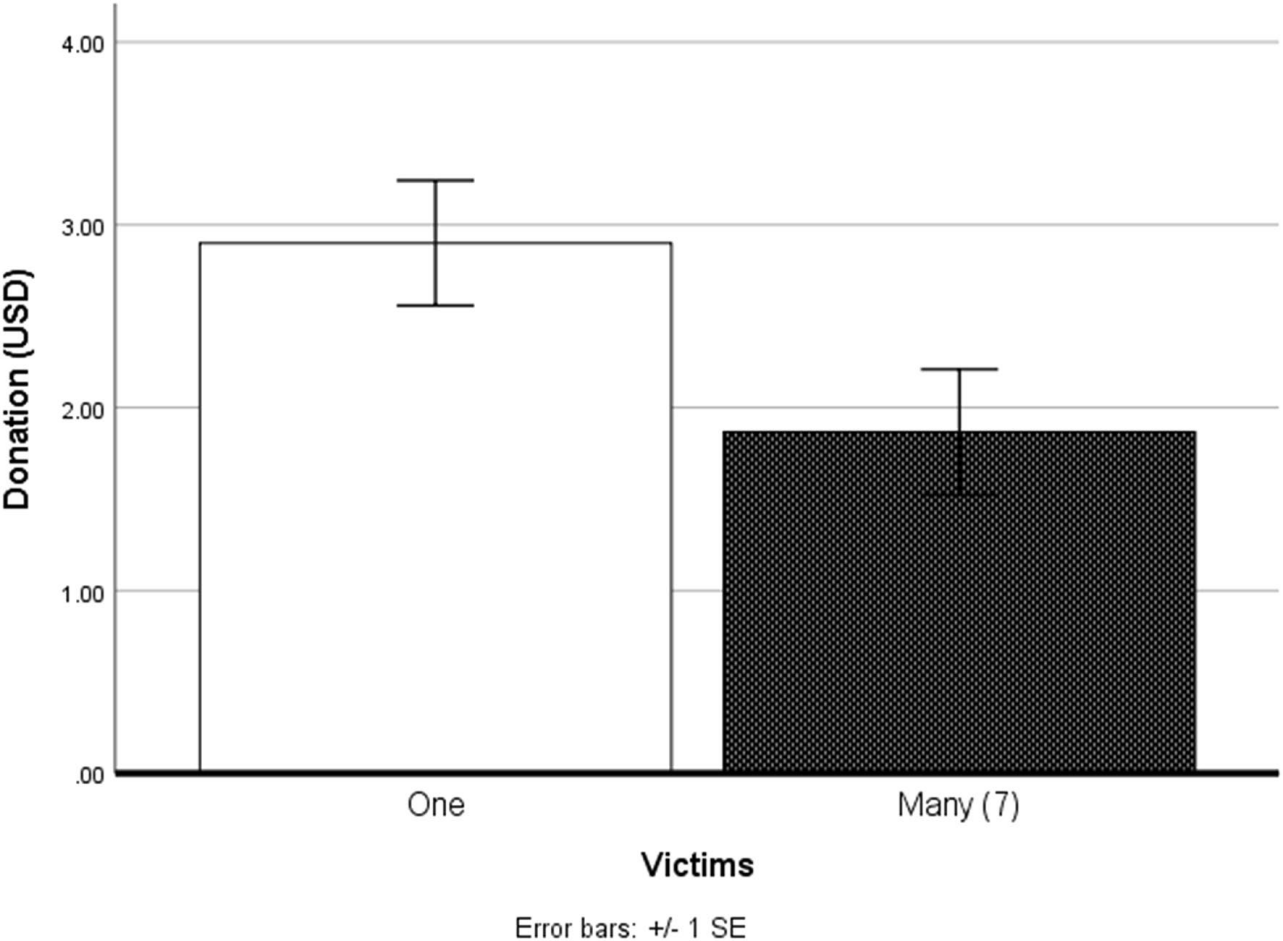
Compassion fade effects observed in Study 1. Participants exposed to a single non-human victim in VR (left) donated significantly more towards sea turtle conservation than those who were exposed to seven victims (right).

As expected, compassion fade was observed among our sample (Fig. 2), demonstrating how victim count in immersive depictions of environmental threats can influence human desire to mitigate those threats. Individuals who viewed the PSA with seven victims donated considerably less towards sea turtle conservation compared to those who viewed the PSA with one victim, which is in line with previous work in non-immersive messaging^6^. To examine how embodying non-human victims may influence compassion fade, Study 2 investigates the requirements for BT with a non-human victim’s (sea turtle) body.

## Study 2: embodying non-human victims in VR

An individual’s body schema is maintained by neural networks that continuously map the body and its interaction with the environment^66^. The malleability of the human corpus is such that one can experience illusory ownership over an artificial limb if that limb (e.g., a rubber hand) is synchronously stroked with one’s hidden real hand (visuotactile synchrony)^67^, or if its movements visually synchronize with the person’s real-world limb movements (visuomotor synchrony)^68^. These principles comprise the rubber hand illusion (RHI) paradigm and can be extrapolated to explain how individuals may come to inhabit a virtual body and integrate it into their self-schema (BT), which ultimately influences how one perceives threats affecting their virtual body^57,69,70^.

Despite growing interest in BT and its effects, the requirements for establishing BT with non-human bodies remain ambiguous. For one, the majority of studies testing the effects of embodiment have done so using humanoid avatars^46,52,71,72^. Second, while the style (or realism) of one’s virtual body does not necessarily detract from BT^73^, morphological similarity to one’s body can influence on BT^73,74^. In the case of animal embodiment, one can identify the incompatibility of non-humanoid avatars, and how this may impede visuomotor representation (e.g., How does one control a virtual tail?). Yet, despite scant literature on animal embodiment, studies suggest that visuotactile stimulation (synchronous haptic feedback) may compensate for incongruent visuomotor feedback associated with embodying an avatar with a unique morphology^75,76^. Thus, Study 2 sought to clarify the individual and combined effects of visuomotor representation and visuotactile stimulation on BT with a sea turtle body in VR (animal embodiment).

### Methods: study 2

**Figure 3:**
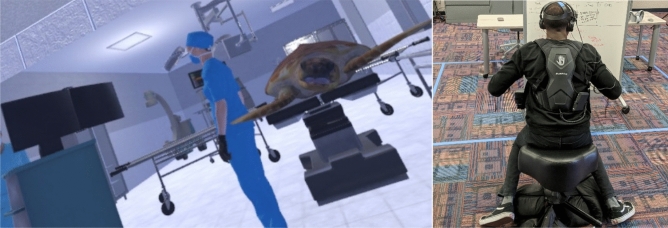
View from the participant’s perspective during animal embodiment. Participants sit on a custom-built seating apparatus allowing for a non-bipedal posture. A SUB-PAC delivers synchronous haptic feedback to the user’s spine, functioning as their carapace in the simulation (right). Users begin the simulation in a virtual sea turtle hospital. Participants in the visuomotor representation condition see, via a virtual mirror, their head and hand movements synchronously reflected onto their sea turtle avatar (left).

A 2 (visuomotor representation: representation vs. no representation) × 2 (visuotactile stimulation: stimulation vs. no stimulation) between-subjects controlled lab experiment tested the impact of the two primary sensorimotor contingencies on BT with a sea turtle body using a custom-built VR installation about sea turtle conservation (Fig. 3). Participants were randomly assigned to one of four versions of a VR simulation about sea turtle conservation. In the visuomotor representation conditions, participants either embodied a loggerhead sea turtle and saw their physical movements reflected onto their sea turtle avatar (representation), or they were disembodied (no representation). In the visuotactile stimulation conditions, participants either received haptic vibrations on their bodies corresponding to in-game events (stimulation), or they did not receive haptic feedback at all (no stimulation).

#### Participants

Participants (*N* = 98; *M*_*age*_ = 20.63, *SD* = 2.71) consisted of college students who were provided course credit in exchange for their participation. Of the 98 participants, 22.4% identified as male (*N*_*male*_ = 22), 75.5% identified as female (*N*_*female*_ = 74), and 2% identified as other (*N*_*other*_ = 2).

#### Stimulus materials

A VR installation (Fig. 3) was developed using Unity 3D software for use with Oculus HMDs (e.g., Oculus Quest, Oculus Rift), a custom-built seating apparatus to allow for non-bipedal posture while embodying a sea turtle, and a haptic feedback backpack (SUB-PAC). The SUB-PAC is a wearable, physical audio system which interfaced with the HMD, interpreting in-game collision information and converting it into deep bass frequencies which transmitted vibrations of varied intensity to the user’s back (see Fig. 3). The installation, titled “Project SHELL (Simulating Living Habitat Experiences of Living Loggerheads)” was created in collaboration with sea turtle experts and allows users to embody a Loggerhead sea turtle at key stages of its life: hatchling, adulthood, nesting. As the user progresses through these stages, they encounter various environmental threats (e.g., marine debris, plastics, boat strikes) and experience their immediate and long-term effects.

#### Story

The version of the simulation used in Studies 2–4 focused exclusively on threats encountered during adulthood, which lasted between 2–5 min. Upon equipping the HMD, hand controllers, headphones, and SUB-PAC, participants emerge in a sea turtle hospital with two nurses, one of whom informs the user that they have been rehabilitated after a boat strike and are set to be reintroduced into the ocean. Afterwards, users are taken to a water tank where users familiarize themselves with the locomotion. The tutorial scene teaches users how to engage in a breaststroke motion to move in the direction they are facing. After learning the swimming mechanics, participants emerge in a virtual coral reef where they are tasked with finding food scattered throughout the coral reef. Users must also resurface occasionally to refill their oxygen. During this experience, participants must avoid various threats, including oncoming boats, netting, marine debris, and microplastics. After three deaths, participants re-emerge in the sea turtle hospital and are informed that the simulation has ended.

#### Visuomotor representation

Participants in the visuomotor representation condition saw their body movements (i.e., head rotation, arm movements) synchronously reflected onto their virtual avatar (i.e., the 3D model of the loggerhead sea turtle). Furthermore, to ensure proper induction of BT with one’s sea turtle body, at the onset of the simulation, users would see themselves in a virtual mirror embodying the turtle avatar prior to beginning the simulation, a method used in previous BT studies^77^. Participants in the no visuomotor representation conditions were in a disembodied state, with only their hand controllers visible throughout the experience.

#### Visuotactile stimulation

Synchronous haptic feedback was delivered throughout the simulation via the use of a haptic backpack (SUB-PAC). For example, at the onset of the experience, the virtual nurse strokes the user’s carapace, sending synchronous haptic vibrations to the SUB-PAC. Additionally, while swimming, users feel a steady vibration representative of friction associated with moving through water. The amplitude of the vibration spikes when the user encounters various threats, as when they are caught in netting or hit by a boat. Participants in the visuotactile stimulation condition received these haptic signals throughout the experience. Participants in the no visuotactile stimulation conditions did not receive haptic feedback at any point. However, these participants were strapped to the SUBPAC during the simulation, though they were informed that the device’s purpose was to improve the tracking of the HMD and the user’s body movements, serving as another sensor to ensure accurate body positioning and movement throughout the simulation.

#### Dependent variables

As in Study 1, environmentalism was included as a control variable, with attitudes, spatial presence, and copresence also assessed. Additionally, four new variables were included: BT, inclusion of nature in self (INS), threat perceptions, and conservation behavioral intentions. BT was measured via a 15-item, 7-point Likert scale assessing participants’ level of agreement with various statements about their virtual representation^26,42^. INS was used to assess the extent to which an individual includes nature into their cognitive representation of self^78^. Previous work suggests that changes in INS can influence conservation-related outcomes, such as involvement with the environmental issue and intentions to mitigate its negative impacts on the environment^26^. INS was measured via a 1-item, 7-point pictorial scale demonstrating a series of seven overlapping Venn diagram circles, with one circle representing the user and the other circle representing nature. Environmental threat perceptions were measured via a 7-item, 7-point (1–7) Likert scale^79^. Lastly, donation intentions consisted of the reported likelihood that the user would donate money towards sea turtle conservation. All scales exhibited moderate to high reliability (all Cronbach’s α > 0.75).

### Results: study 2

**Figure 4:**
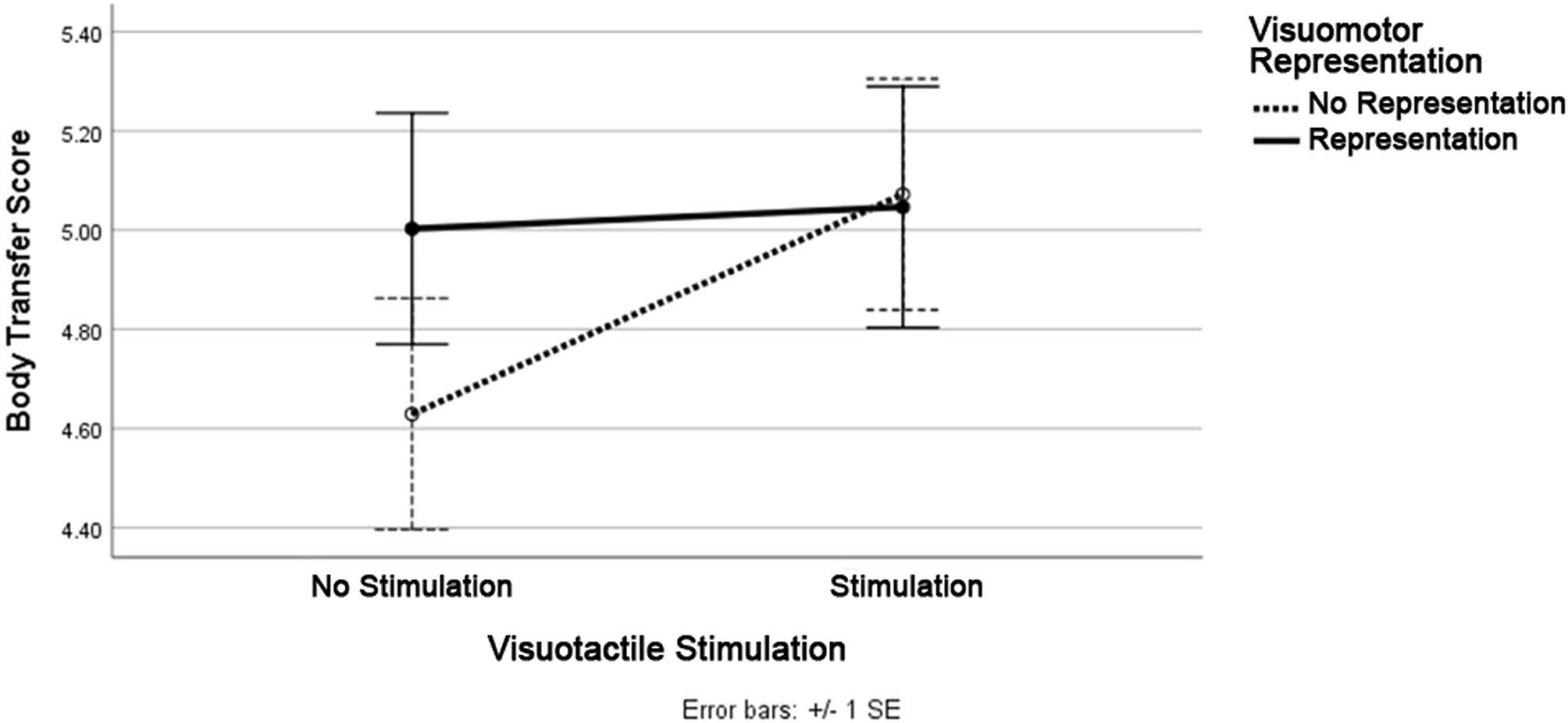
Body transfer scores across the four experimental conditions in Study 2.

A series of two-way ANOVAs revealed no significant differences in self-reported concern for environmental issues (*P* > 0.05), spatial presence (*P* > 0.05), and copresence (*P* > 0.05) across both the visuomotor representation groups. With regards to BT, there were no main effects of visuomotor representation, *F*(1, 94) = 0.54, *P* = *ns,* or visuotactile stimulation, *F*(1,94) = 1.06, *P* = *ns*, on BT (*M*_*BT*_ = 4.93, *SD* = 1.16). Furthermore, there was no significant interaction between visuomotor representation and visuotactile stimulation on BT, *F*(1,94) = 0.72, *P* = *ns*. Thus, all participants experienced a moderately strong sense that they were embodying a sea turtle body (Fig. 4). Lastly, a series of two-way ANOVAs revealed no significant differences in terms of time (minutes) spent in the experiment across the experimental conditions (M = 24.37, SD = 4.35).

A bootstrapped multiple mediation analysis with 5,000 resamples^80^ was conducted, using the PROCESS path-analysis macro (Model 6) in SPSS, to examine whether the effects of BT on donation intentions were mediated by INS and threat perceptions. BT significantly predicted INS (b = 0.53, *P* < 0.001), though INS did not significantly impact donation intentions (b = 0.12, *P* = *ns*). Thus, INS did not mediate the effects of BT on donation intentions. With regards to threat perceptions, BT was a significant predictor of threat perceptions (b = 0.23, *P* = 0.007), and threat perceptions subsequently significantly contributed towards donation intentions (b = 0.54, *P* < 0.001). In sum, the effects of BT on donation intentions towards sea turtle conservation were partially mediated by threat perceptions (b = 0.12, BootSE = 0.12, BootCI = 0.02 to 0.27), but not INS (b = 0.05, BootSE = 0.06), 95% BootCI [0.08, 0.18]. BT remained a significant predictor of donation intention despite having threat perception included in the model (b = 0.51, SE = 0.14), 95% BootCI [0.24, 0.78]. Overall, the results lend support to the notion that embodying a non-human victim increases the salience of the threats to the group, which thereby contribute to charitable giving intentions (Table 2).

**Table 2:** Linear regression analyses of body transfer on key dependent variables in Study 2 across experimental conditions.

	*R*^2^	*B*	*SE B*	*β*	*t-stat*
**BT → INS**					
No Visuomotor Representation/No Visuotactile Stimulation (N = 25)	0.29	0.17	1.07	0.54	3.09**
Visuomotor Representation/No Visuotactile Stimulation (N = 25)	0.19	0.61	0.25	0.44	2.36*
No Visuomotor Representation/Visuotactile Stimulation (N = 25)	0.13	0.42	0.22	0.36	1.86
Visuomotor Representation/Visuotactile Stimulation (N = 23)	0.11	0.34	0.21	0.33	1.62
**BT → Threat Perceptions**					
No Visuomotor Representation/No Visuotactile Stimulation (N = 25)	0.01	0.06	0.17	0.07	0.33
Visuomotor Representation/No Visuotactile Stimulation (N = 25)	0.22	0.37	0.14	0.47	2.58*
No Visuomotor Representation/Visuotactile Stimulation (N = 25)	0.11	0.33	0.19	0.34	1.71
Visuomotor Representation/Visuotactile Stimulation (N = 23)	0.05	0.21	0.2	0.22	1.02
**BT → Donation Intentions**					
No Visuomotor Representation/No Visuotactile Stimulation (N = 25)	0	0.07	0.29	0.06	0.26
Visuomotor Representation/No Visuotactile Stimulation (N = 25)	0.21	0.94	0.38	0.46	2.46*
No Visuomotor Representation/Visuotactile Stimulation (N = 25)	0.17	0.51	0.23	0.42	2.19*
Visuomotor Representation/Visuotactile Stimulation (N = 23)	0.49	0.98	0.22	0.7	4.53***

*P < 0.05, **P < 0.01, ***P < 0.001.

### Discussion: study 2

Phenomenologically, six factors are identified as determinants of BT: body ownership, agency, haptics, body location, body appearance, and body responses to stimuli^81^. The null effects of the visuomotor and visuotactile conditions discount the theory of a compounding effect of visuotactile stimulation and visuomotor representation, running contrary to literature showing that visuomotor factors are a greater contributor to BT than visuotactile factors, and that the disruption of either can contribute to a break in the illusion^42,75,81^. The results also run counter to previous work finding that embodiment of an avatar, regardless of the degree of realism, elicits higher BT than having no avatar^82^. Slater and colleagues have noted that there is uncertainty around whether the illusion would hold if there were structural changes to the body^42^. The results also disambiguate this concern by demonstrating that, regardless of visual representation, BT with a non-bipedal creature is achievable.

The null effects of visuomotor representation and visuotactile synchrony on BT also suggest that proprioceptive factors (e.g., non-human locomotion), may have overridden the individual effects of these components. Even those without a virtual body assumed the posture, movement patterns, and morphology (e.g., carapace proxy via the haptic backpack) of the non-human creature. Previous work argues that body representation may not be present and still elicit embodiment when individuals hold an unconscious assumption that something similar to their regular body (image) persists in a given virtual environment^83^. Thus, perceptual judgments of one’s body are differentially impacted by the combination of movement, visual, and haptic factors.

Alternatively, the null differences may be rooted in properties specific to how haptic feedback was delivered. Participants experienced a sustained haptic vibration while underwater (mimicking water friction), which spiked in frequency (strength) during collisions with environmental threats. The baseline vibrations (water friction) may have habituated users to haptic sensations and created a ceiling effect. Indeed, previous studies examining synchronous sporadic versus consistent haptic feedback during embodiment^26^ lend support to this notion, though future work is needed to support this theory.

While BT significantly predicted INS and threat perceptions, only the latter subsequently contributed towards donation intentions. Of particular interest is the direct effect of visuotactile synchrony on INS. This finding underscores the affective nature of hapticity: touch is considered “a powerful conduit for emotional connectedness”^84^ (p. 376) capable of influencing human judgment and decision-making^85,86^, even in cases where the source of the haptic information is separate and unrelated to the object being evaluated^87^. Moreover, the valence of the haptic information itself influences judgment formation, as positive (negative) haptic sensory experiences can contribute (detract) from one’s evaluation of an object/experience^88^. However, the haptic information presented in the simulation signaled negative (boat strikes) and neutral (ocean vibrations) information. Given the main effect of hapticity on INS, and the absence of main effects on threat perceptions, BT, and attitudes, it suggests haptic feedback informed judgment of the species, as opposed to the simulation or threat.

## Study 3: the effects of BT on compassion fade

### Methods: study 3

Study 3 explored whether animal embodiment’s influence on users’ affective, cognitive, and behavioral responses to an environmental threat vary as a function of the number of victims (compassion fade). A 3-condition (victims: self vs. one vs. seven) between-subjects field experiment was conducted at a large museum in 2019.

#### Participants

Museum visitors (*N* = 90; *M*_*age*_ = 18.95, *SD* = 16.61) participated in the study voluntarily without compensation. Of the 90 participants, 56.7% identified as male (N_male_ = 51) and 43.3% identified as female (N_female_ = 39).

#### Stimulus materials

**Figure 5:**
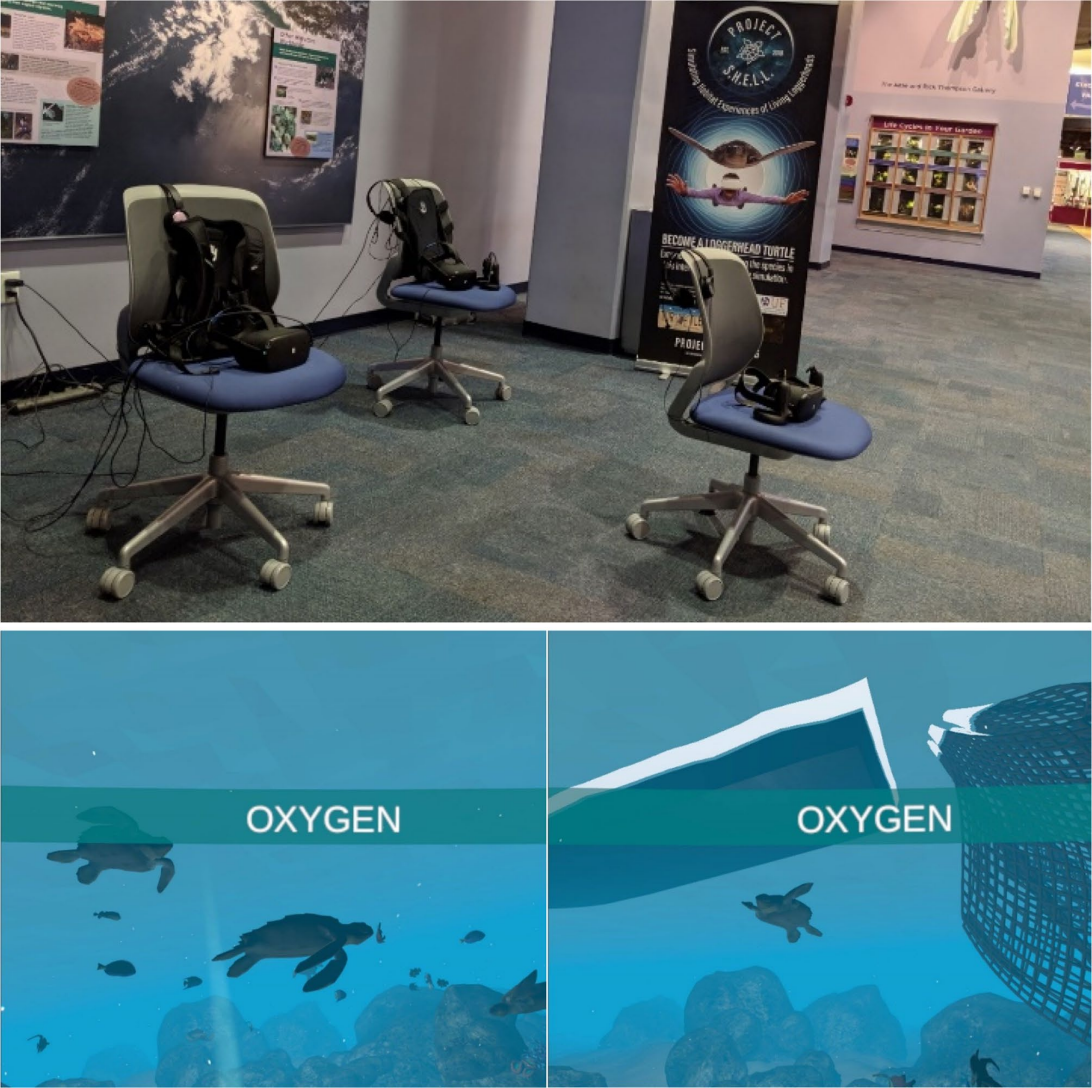
Experimental set-up and stimuli used in Study 3. Field study participants were randomly assigned to one of three VR stations (top), each representing one of three experimental conditions. In-game screenshots demonstrate the salience of victim count (seven: bottom left), and of particular environmental threats, such as getting caught in fishing netting (bottom right).

A designated area in the museum was provided for the Project SHELL installation, which consisted of three chairs, each with a SUBPAC haptic backpack, an Oculus Quest, and a dedicated researcher to assist with on/offboarding (Fig. 5). Each HMD was pre-loaded with one of three modified versions of the simulation. The narrative structure of Project SHELL remained consistent: users embody a rehabilitated Loggerhead in a medical facility, are reintroduced into the ocean where they encounter various threats and are returned to the hospital upon completion. Users could complete the simulation in one of three ways: 1) after dying three times, 2) after eating all of the food in the environment, or 3) after surviving for five minutes. The victim count manipulation was made salient in all three sequences; users would either be the lone sea turtle (self-as-victim; N = 29), see one other sea turtle (N = 27), or see seven other sea turtles (N = 34). Thus, one version of the simulation projected the threats solely onto the user, whereas the other two versions place either one or seven victims alongside users. Behavior of the sea turtles (victims) was programmed within Unity to mimic natural navigation and collision avoidance within the ocean scene. Lastly, due to facility restrictions and liability, the seating apparatus used in Study 2 was replaced with a swivel chair (see Fig. 5).

#### Procedures

Project SHELL was promoted by the museum as a temporary installation, with signage and digital communications informing visitors of the opportunity to test a VR experience about sea turtles. Visitors who approached the installation were given information about Project SHELL and the opportunity to participate in the unpaid study which consisted of using the simulation and completing an online questionnaire afterwards. However, visitors were also given the opportunity to test the simulation without participating in the study. Visitors who agreed to participate were provided with an informed consent form to sign. For children, parental consent forms were provided and signed prior to participation. After an onboarding process, participants finished the simulation and subsequently completed a post-questionnaire measuring variables of interest.

#### Dependent variables

Due to the nature of the field study, namely time constraints and the need to minimize line queues, several alterations were made to the post-questionnaire. Several survey items were omitted and/or modified, with the exception of BT and INS, which were retained and deployed in their original forms. Environmentalism and threat perception scales were removed due to length. Instead, a single item measure of perceived extinction was used as a proxy (see below for a detailed description of new measures included in Study 3). Attitudes towards the simulation, which were previously measured via a multi-item Likert scale, were instead assessed via a star rating system (1–7). Lastly, donation intentions were measured via a hypothetical scenario^89^. Participants were asked to imagine winning $50 and list how much they would keep, and how much they would donate towards Loggerhead sea turtle conservation efforts. This behavioral intention outcome was deemed appropriate considering that hypothetical donation behaviors have been shown to correlate with real donation behaviors in environmental communication contexts^89^. All scales exhibited moderate to high reliability (all Cronbach’s α > 0.75).

### Results: study 3

A one-way ANOVA revealed that the manipulation of victim amount was successful. Participants’ estimates of the number of sea turtle sencountered in the simulation varied significantly across the three groups, *F*(1,87) = 24.81, *P* < 0.001. One-way ANOVAs also failed to detect main effects of victim count on attitudes (*F*(1,87) = 1.84 *P* = *ns*), BT (*F*(2, 89) = 0.22, *P* = *ns*), INS (*F*(2,87) = 1.04 *P* = ns), or threat perceptions (*F*(2,87) = 2.3, *P* = *ns*). Mean scores for all measured variables across the three experimental groups are shown in Table 3.

**Table 3:** Mean scores across experimental conditions in Study 3.

	N	Mean	SD
**Self (no victims)**			
What is your age (in years)?	29	17.33	15.47
Have you used VR before?	29	1.55	0.51
Time in VR	29	6.34	2.72
Fish encountered	26	23.65	18.98
Turtles encountered	29	.34	0.55
Perceived interactivity	29	6.10	1.45
INS	29	4.93	1.94
Threat perceptions	29	4.07	1.56
Stars	29	6.24	1.05
Donation	29	29.00	16.10
Body transfer	29	4.79	1.14
**One victim**			
What is your age (in years)?	27	16.30	14.08
Have you used VR before?	27	1.70	0.47
Time in VR	27	7.11	2.49
Fish encountered	25	21.64	17.89
Turtles encountered	27	3.74	6
Perceived interactivity	27	6.67	1.21
INS	27	5.33	1.59
Threat perceptions	27	3.85	1.59
Stars	27	6.56	0.69
Donation	27	34.59	16.48
Body transfer	27	4.78	1.14
**Seven victims**			
What is your age (in years)?	34	22.44	19.08
Have you used VR before?	34	1.53	0.51
Time in VR	34	7.06	2.39
Fish encountered	33	20.97	15.07
Turtles encountered	34	10.09	7.33
Perceived interactivity	34	6.35	1.30
INS	34	5.53	1.44
Threat perceptions	34	4.65	1.41
Stars	34	6.12	0.91
Donation	34	29.85	19.17
Body transfer	34	4.61	1.36

#### Compassion fade

**Figure 6:**
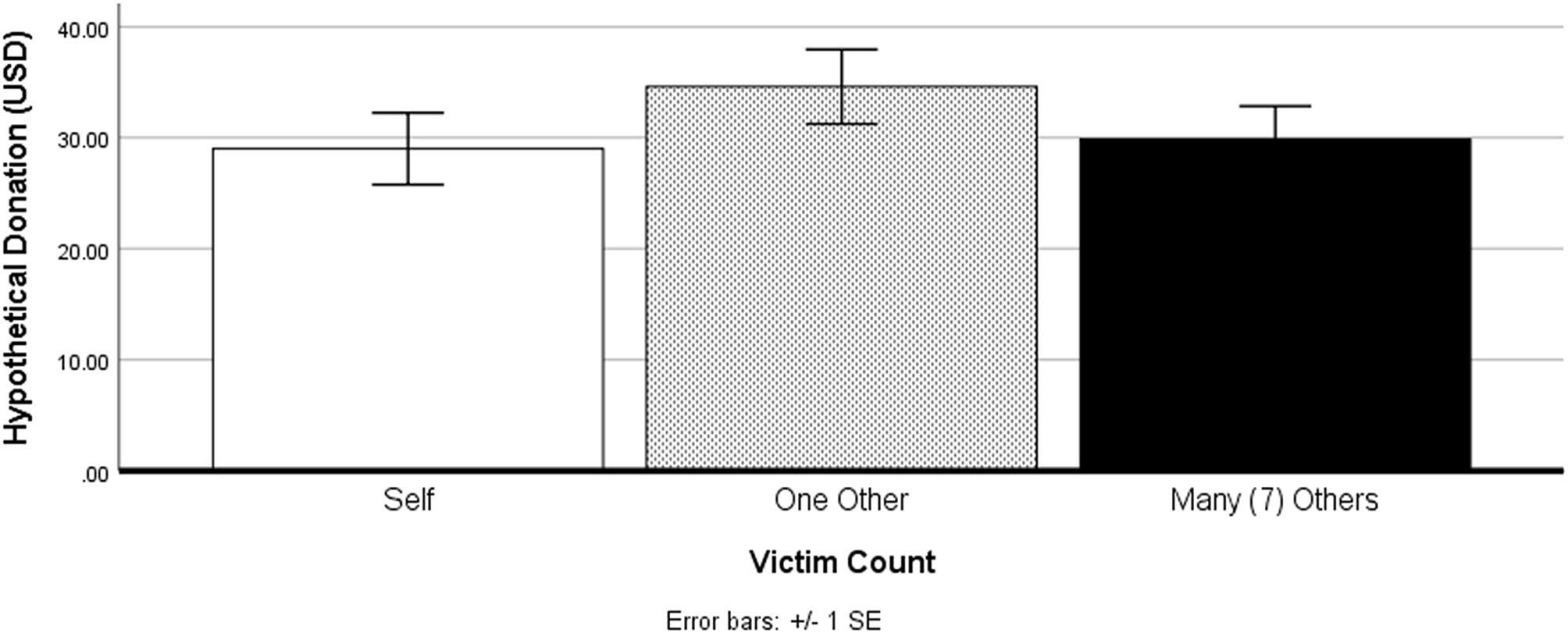
Differences in hypothetical sea turtle conservation donations across experimental groups in Study 3. There were no statistically significant differences in hypothetical donations across experimental conditions.

There were no main effects of victim amount on the hypothetical donations, *F*(2,87) = 0.84, *P* = ns. Moreover, there were no significant differences in pledged donation amounts between the seven (M = 29.85, SD = 19.16) and single victim condition (M = 34.59, SD = 16.47), *P* = ns. The results suggest that exposure to mass casualty while embodying a victim can inhibit compassion fade effects (see Fig. 6).

A linear regression analysis demonstrated that BT significantly predicted hypothetical donations, *R*^2^ = 0.05, *β* = 3.27, *SE* = 1.48, *P* = 0.03. If indeed compassion fade was at-play during embodied exposure to mass casualty, victim amount should moderate the effects of BT on donation intentions. A bootstrapped multiple mediation analysis with 5,000 resamples^80^ was conducted, using the PROCESS path-analysis macro (Model 1) in SPSS, to examine whether the effects of BT on hypothetical donations were moderated by victim amount. BT significantly predicted donation intentions (b = 9.37, *P* = 0.025). However, there was no significant interaction between BT and victim amount (*b* = -2.77, *P* = ns), R^2^∆ = 0.02, *F*(1,86) = 2.48, *P* > 0.05. In sum, there was no evidence of compassion fade in scenarios where audiences (a) embody the victim, and (b) encounter seven other victims versus a single identifiable victim.

To examine whether BT influenced hypothetical donation amount through mechanisms proposed in Study 2, a bootstrapped multiple mediation analysis with 5,000 resamples^80^ was conducted using the PROCESS path-analysis macro (Model 6) in SPSS; The model tested whether the effects of BT on donation intentions were mediated by increased INS and threat perceptions (perceived extinction classification). As in Study 2, BT significantly predicted INS (b = 0.67, *P* < 0.001), though INS did not significantly influence donation intentions (b = -0.73, *P* = *ns*). Thus, INS did not mediate the effects of BT on donation intentions. With regards to threat perceptions, BT was not a significant predictor of threat perceptions (b = -0.05, *P* = *ns*), nor did threat perceptions significantly contribute towards donation intentions (b = -0.56, *P* = *ns*). In sum, the effects of BT on donation intentions towards sea turtle conservation were neither mediated by INS (b = -0.49, *BootSE* = 0.82) 95% BootCI [2.13, 1.17] nor perceived extinction classification (b = 0.02, *BootSE* = 0.25) 95% BootCI [-0.35, 0.69], though BT remained a significant predictor of donation amount (b = 3.27, *SE* = 1.48) 95% BootCI [0.32, 6.21]. Overall, the results lend support to the notion that embodying a victim increases the salience of the threats to the group, which can thereby increase altruistic behavioral intentions.

**Figure 7:**
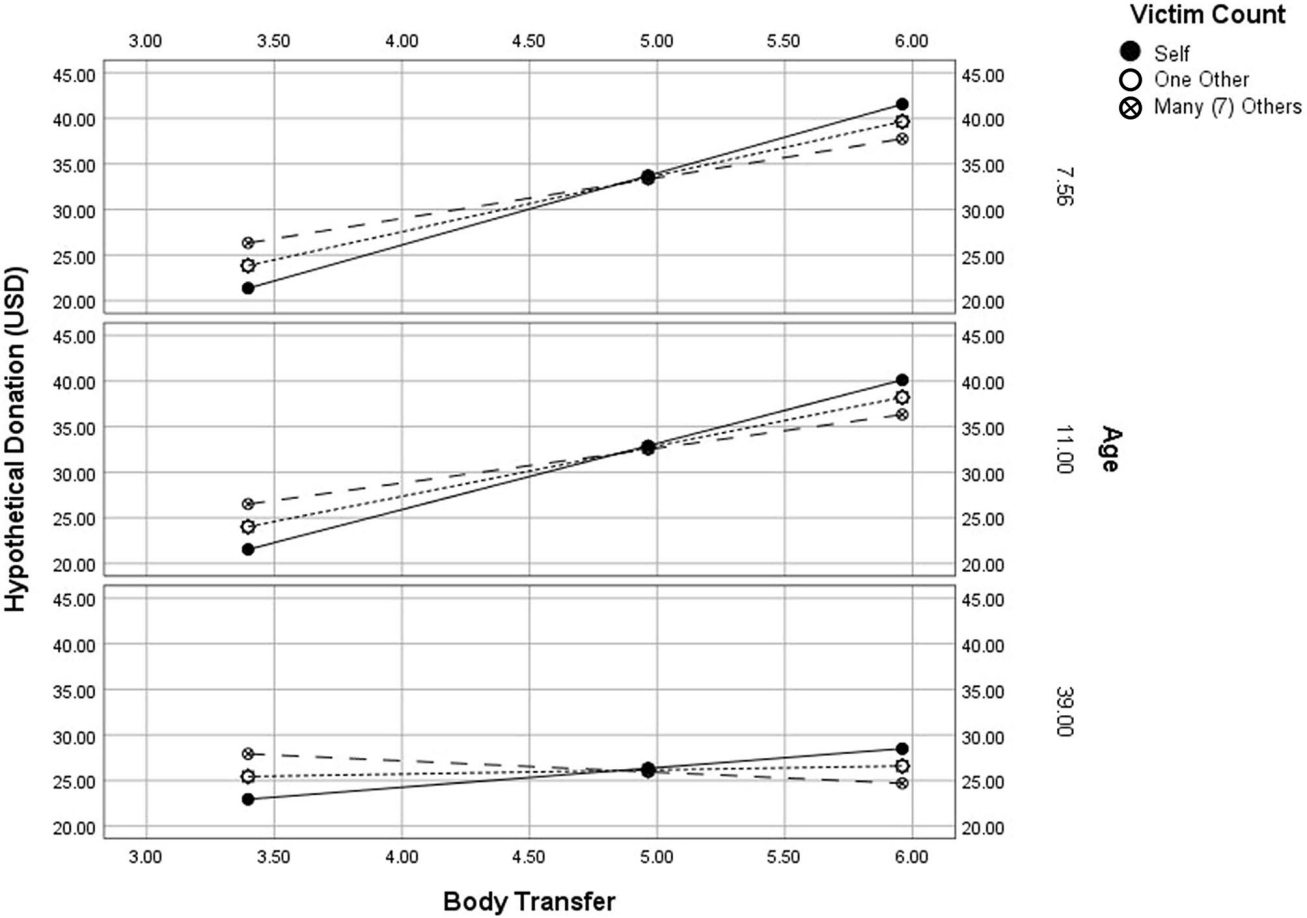
The differential effects of body transfer on donation amount as a function of age and victim count. Age significantly moderated the effects of body transfer on donation amount.

Due to the large variance and range in participants’ ages, and the nature of the BT illusion, it is imperative to account for developmental differences in the sample. Of particular importance is recent work suggesting that, as humans progress through childhood and develop their own-body perception, people become less sensitive to illusory BT^90^. A moderation analysis was implemented using the Process Macro (Hayes, 2015; Model 1) with BT as the predictor, age as the moderator, and hypothetical donation amount as the dependent variable. BT significantly predicted hypothetical donation amounts (b = 7.73, SE = 2.14, *P* < 0.001). Furthermore, there was a significant interaction between BT and age (b = -0.201, SE = 0.07, *P* = 0.003) such that BT’s positive influence on donation amount diminished with age (Fig. 7).

### Discussion: study 3

Studies examining compassion fade have established that individuals contribute more real and hypothetical resources towards ensuring the well-being of single rather than several victims^91^. The results suggest that compassion fade can be offset at least in part by embodied perspective-taking. Individuals who embodied a virtual loggerhead turtle and experienced threats alongside seven turtles did not differ in terms of hypothetical donations toward sea turtle conservation compared to those who experienced the same simulation alongside one victim or no victims.

As BT served as a significant direct predictor of giving behavior regardless of victim count, the extent to which individuals feel a non-human victim’s body is their own is a viable mechanism for offsetting compassion fade. Yet, the inability to replicate Study 2’s partial mediation lends credence to cautionary notes about the interpretation of mediators (e.g., INS) in the context of BT^49^. A growing body of literature argues that mediation pathways, particularly in an experimental context, may be difficult to establish in a single study^92^. Indeed, when coupled with the fact that BT significantly (and directly) contributed towards giving behavior in both Study 2 and Study 3, it can be argued that the self-other overlap instantiated by BT is a mechanism for altruistic behavior in and of itself.

Identifying why BT, but not connectedness with the species (INS), influences hypothetical giving requires identifying key distinctions between BT and INS. INS measures the sense of connectedness to another species, whereas BT measures the psychological sense that one has become an exemplar of that species. A connection to nature is considered global concept affected by many factors^93^, encapsulating an enduring, high-level relationship between humans and broad aspects of nature (i.e., entire species or biomes). Conversely, BT is characterized by the immediacy and salience of the sensory experience as the virtual body. The absence of granularity in the INS measure limits the ability to parse out the impact of episodic experiences on these high-level assessments of the human-species dynamic. Thus, while BT may engender closeness with the species, this closeness may not dictate subsequent processing of the threats and the motivation to mitigate them in the short term through charitable giving.

The non-significant indirect effect of BT on giving behavior through threat perceptions also raises questions about the instrument used to assess perceived threat severity. Whereas the threat perception scale in Study 2 assessed future impacts of various threats on the survivability of the species, Study 3 measured participants’ perceptions of the species’ current survivability. It can be argued that it is general knowledge that Loggerhead sea turtles are not extinct, which would skew estimations. Additionally, because the experimental manipulation involved the presence of several turtles (versus none or one), participants may have taken the seven victims condition as a heuristic of a thriving species. The encountered threats may indeed have been perceived as more severe due to experiencing them alongside other similar victims, though the extinction classification measure may have failed to capture this heightened severity.

Another important insight relates to the moderating role of age. Previous work has demonstrated that adults are less susceptible to body ownership illusions^90^. Study 3 extends this work to include non-human body schema as well: the strength of the BT illusion diminished as age increased. While not the primary focus of this investigation, the moderating effects of age emphasizes an important insight for facilities like zoos and aquariums which provide immersive experiences for children and adults. Whereas mere embodiment may suffice in inspiring youth to take action, it may be that message factors (e.g., source credibility, gamification elements) may be better suited for older adults. The potential influence of age on responses to subjective measures should also be noted.While Study 3 relied primarily on pictorial scales, which have been used in previous environmental studies with young children^94^, hypothetical giving behavior may have been influenced by age. Previous work suggests that altruistic behavior (e.g., monetary giving) varies according to age^95^. However, given that age was evenly distributed across the experimental groups, it is unlikely that age significantly altered our results. Future work should continue to explore the interplay between demographic, technological, and message factors in the context of immersive conservation experiences like Project SHELL.

## Study 4: examining the interplay between BT, victim similarity, and compassion fade

### Methods: study 4

Study 3’s results establish support for the notion that embodying non-human victims can offset compassion fade, though whether this effect is driven by connectedness with the victimized group or heightened threat salience remains to be seen. To address this, a 2 (victims: one vs. seven) × 2 (victim species: similar vs. dissimilar) between-subjects experiment was conducted in the same controlled lab setting as with studies 1 and 2.

#### Participants

Twenty-five university students (N = 25) were recruited using an undergraduate research pool and were paid $5 in exchange for their participation. The mean age of participants was 21.44 (SD = 3.42). Of the 25 participants, 12% identified as male (N_male_ = 3) and 88% identified as female (N_female_ = 22).

#### Stimulus materials

As previously mentioned, the Project SHELL simulation used in Study 3 served as the foundational basis for the simulations used in Study 4. Specifically, four (4) different versions of the experience were ported to the Oculus Quest HMD, each representing one of the four experimental conditions. Five participants were randomly assigned to the one similar victim condition, six to the one different victim condition, eight to the seven similar victims condition, and six to the seven different victims condition. As in Study 2, participants used the simulation with the custom-designed chair. Major modifications to the content itself pertained to the introduction of the new victim species (i.e., bottlenose dolphins) elaborated upon below. In all conditions, users embodied a Loggerhead sea turtle.

To create a plausible yet salient exposure to dissimilar victims, Study 4 implemented modifications to the simulation. As in Study 3, exposure to victims were made salient at three key points in the linear embodied experience: (1) upon emerging in the hospital environment, (2) in the ocean environment, and (3) upon returning to the hospital environment. The major modification of these simulations involved swapping the loggerhead sea turtle models of the single and seven victims with that of another marine species: The bottlenose dolphin.

The bottlenose dolphin was chosen as the dissimilar species for several reasons. First, sea turtles may plausibly encounter other dolphins in the wild. Second, the dolphin is of a similar size as the loggerhead sea turtle, though it exhibits ample visual differences which make salient the species’ aesthetic uniqueness from the user’s embodied species (sea turtle). Third, both species face similar threats and thus may be admitted to the same marine rehabilitation centers. However, because the hospital setting used in previous versions of the simulation (Studies 1–3) were framed as taking place in The Turtle Hospital, which does not rehabilitate dolphins on-site, another organization (Mote Marine Laboratory) had to serve as the host center. The 3D dolphin models were acquired via the Unity 3D asset store, and appropriate animations were adapted for use with these models.

#### Procedures

The procedure of this study was identical to that of Study 2 except for the inclusion of the donation behavioral measure included at the end of the study. This donation behavioral measure was implemented using the same procedure from Study 1, with one major exception: participants were told that any donations given from their $5 would go towards the Mote Marine Laboratory and their conservation efforts, as opposed to The Turtle Hospital in Study 1.

#### Dependent variables

This study employed the same dependent and control variables as used in Study 2, and the inclusion of the donation behavioral measure as used in Study 1. Additionally, given time limitations in Study 2, perceived species extinction classification served as a proxy for threat perceptions. To assess whether this served as a valid substitute, it was included in this study alongside the original threat perceptions scale used in Study 2. All scales exhibited moderate to high reliability (all Cronbach’s α > 0.75). Lastly, to control for potential differences in species favorability, participants were asked to rate how much they cared about various marine species, including sea turtles and dolphins.

### Results: study 4

**Figure 8:**
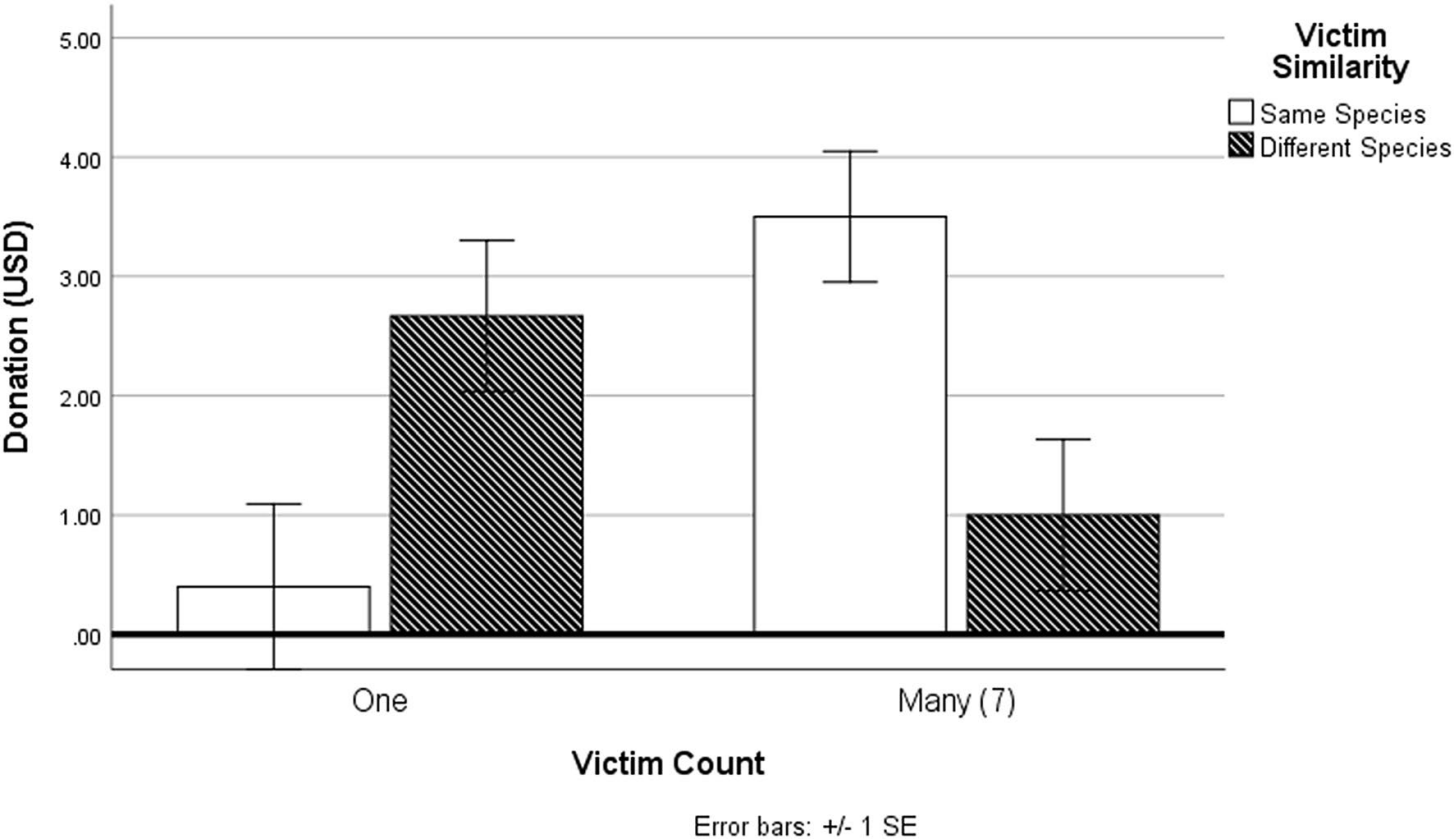
Differences in charitable giving as a function of victim count and victim similarity. Compassion fade was observed when victims were of a different species than the user, though this effect was reversed when victims were of the same species.

A series of two-way ANOVAs and associated pairwise comparisons found no significant main effects of victim count or victim similarity on all dependent variables with the exception of actual donations. A two-way interaction effect between victim amount and victim similarity on donation amount was predicted. To test this assumption, a two-way ANOVA was conducted with victim amount and victim similarity as two independent variables, and donation amount as the dependent variable. The results demonstrated a significant two-way interaction, *F*(1,21) = 14.34, *P* = 0.001, such that compassion fade was observed for dissimilar victims as opposed to similar victims (see Fig. 8). Users who encountered several dissimilar victims (*M*_*SevenDolphins*_ = 1, *SD* = 2.06) donated less towards marine conservation compared to those who encountered a single dissimilar victim (*M*_*OneDolphin*_ = 2.67, *SD* = 2.06), indicative of psychic numbing, *F*(1,21) = 3.46, *P* = 0.07, partial ή^2^ = 0.14, *d* = 0.99, though this effect was non-significant. This effect was reversed for those encountering victims of the same species; participants who encountered many similar victims (*M*_*SevenTurtles*_ = 3.5, *SD* = 1.77) donated significantly more towards marine conservation compared to those who encountered a single similar victim (*M*_*OneTurtle*_ = 0.4, *SD* = 0.55), *F*(1,21) = 12.28, *P* = 0.002, partial ή^2^ = 0.4, *d* = 2.36.

A series of linear regressions were conducted to examine the relationship between BT and connectedness with each specific victim species (INS). Among participants presented with dissimilar victims, BT was not a significant predictor of connectedness with bottlenose dolphins, *R*^2^ = 0.09, *β* = -0.38, SE = 0.38, *P* = *ns.* However, among participants presented with similar victims, BT was a significant predictor of connectedness with loggerhead sea turtles, *R*^2^ = 0.49, *β* = 0.7, SE = 0.22, *P* = 0.008. Lastly, across the entire sample, BT was a significant predictor of connectedness with loggerhead sea turtles, *R*^2^ = 0.38, *β* = 0.77, SE = 0.2, *P* = 0.001. Thus, as in Study 2 and Study 3, BT significantly contributed to connectedness with the Loggerhead turtle species.

There were no main effects of victim amount, *F*(1,21) = 0.00, *P* = *ns*, or victim similarity, *F*(1,21) = 0.03, *P* = *ns*, on the perceived extinction classification of loggerhead sea turtles. Furthermore, there were no main effects of victim amount, *F*(1,21) = 0.12, *P* = *ns*, on threat perceptions (Cronbach’s α = 0.78). However, a main effect of victim similarity was observed, *F*(1,21) = 6.76, *P* = 0.017, such that participants perceived there to be more severe threats facing different (*M*_*Dolphins*_ = 5.93, *SD* = 0.79) rather than similar (*M*_*Turtle*_ = 5.26, *SD* = 0.51) victims. A linear regression was conducted to examine the relationship between BT and threat perceptions. The results demonstrated that BT did not significantly predict the perceived probability and severity of environmental threats facing marine life (threat perceptions), *R*^2^ = 0.002, *β* = 0.03, SE = 0.15, *P* = *ns.*

### Discussion: study 4

The human tendency to group other beings into social categories to distinguish one’s self from others is a robust phenomenon in social psychology. This propensity for in/out-group classification may explain a key finding in this study: humans donated more towards marine conservation when they were exposed to several victims of the same species as opposed to one identifiable victim of the same species, whereas the reverse was true for dissimilar species. Humans favor similarity and behave more altruistically towards those with similarity cues^96^. As Tian and Konrath (2019) note, similarity is a social construct integral to shaping attitudes, behaviors, and perceptions^97^. In their systemic review of similarity effects, evidence supports the notion that human donation behavior increases when beneficiaries of giving behavior are similar to the donor across a variety of personal attributes, including those which are temporarily induced (e.g., temporary group membership). Previous work has also suggested that environmental compassion fade may vary as a function of whether animals are considered in-group versus out-group members^6^. The results in Study 4 lend support to the notion that (a) embodying threatened wildlife (i.e., victims) in VR can reverse psychic numbing effects (compassion fade), and (b) that this effect is contingent on victim similarity. Moreover, the finding that donations were greater among those who encountered several similar victims as opposed to one similar victim runs counter to previous work showing compassion fade occurs with in-group members^98^. While pairwise comparisons revealed the mean differences for the dissimilar victim conditions to be non-significant (*P* = 0.07), this is likely due to the study’s sample size and lack of statistical power.

## General discussion

Study 1 confirmed the existence of compassion fade when depicting threatened wildlife in VR. To examine whether embodying threatened wildlife would influence this effect, Study 2 investigated the role of visuomotor representation and visuotactile stimulation in driving BT with a sea turtle body. The results demonstrated that moderate levels of BT with a non-human body are achievable, though the isolated and combined effects of visuomotor representation and visuotactile stimulation in driving that illusion remain ambiguous. It was proposed that visuomotor representation and visuotactile stimulation would, in tandem, undergird the sense of BT. However, the null results ultimately run contrary to this prediction and highlight the nuances of body ownership. Having established a mechanism for non-human embodiment, a field experiment (Study 3) demonstrated that animal embodiment can mitigate compassion fade. Study 4 further established preliminary support for the notion that such mitigation may be contingent on the similarity of the victim to the user’s virtual body. Collectively, the four studies provide modest but rich insights into the underlying mechanisms driving illusory body transfer with non-human bodies, elucidate the psychological and behavioral effects of such embodiment, and highlight how animal embodiment may be leveraged for biodiversity conservation.

A major contribution of the current work relates to clarifying how visuomotor representation and visuotactile stimulation shape BT with virtual bodies of non-human morphologies. Several studies have demonstrated that moderate levels of body ownership can be achieved through visuomotor representation or visuotactile stimulation^68,99^. While some research suggests that haptic feedback can enhance BT^44,100^, other studies suggest that visuomotor synchrony is the primary mechanism for this illusion^43,99^. Acknowledging the importance of both modalities, Spanlang et al. (2014) suggest that multimodal stimulation systems – VR systems that combine synchronous haptic feedback with full body tracking – “can provide a greater degree of the feeling of ownership over the virtual body” compared to unimodal stimulation (p. 10)^101^, and have even been shown to influence perception of other characters in the environment^102^. Extant literature on joint interpretation of visual and haptic data also argues that both types of sensory information merge into a unified perception of the events/scene^103^. The amalgamation of visual, audio, and tactile feedback from the simulation should therefore maximize the degree to which users feel they are embodying the virtual creature. This effect would be in line with previous research which argues that illusory experiences of presence and BT are predicated on a “precisely coordinated synthesis of separate sensory input channels”^104^ (p. 757). Future work must continue to examine these factors during non-human embodiment to determine how to achieve (and maintain) a sense of ownership over a unique body.

A long-standing argument among VR researchers examining the platform’s potential for driving social change is that BT can contribute to prosocial outcomes by increasing empathy for the embodied (human) group^105^. While the capacity for empathy is observed in many mammals^106^, inter-species empathy is less understood. For example, there are various issues surrounding the measurement of empathy for non-human victims, namely the inability for empathy scales to “tap into a single unitary construct”^107^. Proposing BT as a driver of conservation outcomes through empathy-related constructs may also prove redundant. If empathy requires understanding the lived experience of an “other” and contributes to prosocial outcomes^108^, BT may effectively modulate conservation actions independent of the proposed mediators (i.e., INS, threat perceptions). In inducing the BT illusion, users are exposed to the lived experience of the other in more visceral terms; feelings associated with exposure to environmental threats are presumably amplified through the perception that they are actually affecting the user’s corporeal body. In this way, BT relates to empathy constructs in that it documents the degree to which users felt their virtual body was a conduit for the victim’s lived experience, as opposed to being a mediated observation of adverse events. This is further supported by the significant indirect effects of BT on donation intentions through heightened threat perceptions. Yet, the inability for INS to mediate the direct effects of BT on giving behavior adds to the growing body of evidence finding INS to be an inconsistent mechanism for pro-environmental behaviors^109^. This also lends credence to cautionary notes about the interpretation of mediators in the context of BT^49^.
